# Editorial: Protistan phagotrophy and the far-reaching implications

**DOI:** 10.3389/fmicb.2026.1798549

**Published:** 2026-02-18

**Authors:** Milos Legner, Edward Fillery, Miroslav Macek

**Affiliations:** 1 University of Toronto, Toronto, ON, Canada; 2 Universidad Nacional Autonoma de Mexico, Facultad de Estudios Superiores Iztacala, Tlalnepantla de Baz, Mexico

**Keywords:** carbon cycle and sequestration, ciliopathy disease, cilliates, ecosystem models, oceanography analysis, phagotrophy

Phagotrophy is one of the most ancient and consequential biological processes on earth. Once adopted by early unicellular organisms, it fundamentally reshaped ecosystem function by allowing populations to thrive independently of direct access to primary energy sources. This innovation increased ecosystem robustness and left a deep evolutionary imprint: the molecular and cellular machinery of phagocytosis remains conserved in modern multicellular genomes, now more or less tightly regulated and expressed in specialized cells.

The goal of this Topic was to attract manuscripts that illuminate the complexity of aquatic food webs, clarify predator–prey interactions—particularly in deep ocean systems—and highlight the broader implications of protist biology for human health. We were especially interested in contributions that revealed the far-reaching consequences of these processes. From seven submissions, six papers were selected that collectively advance our understanding of microbial ecology, biogeochemical cycling, and evolutionary cell biology.

Quantifying grazer-mediated prey mortality is essential for understanding carbon fluxes in aquatic ecosystems (Strzepek et al., 2022). Building on earlier methodological standardization efforts (Montagnes and Berges, 2004), Weisse provides guidance on optimizing experiments to estimate numerical (NR) and functional (FR) responses of protistan populations. These parameters are based on the Lotka–Volterra (LV) predator–prey model, in which predator growth (NR) is proportional to ingestion rate (FR) (Yang et al., 2013).

Although widely used, the LV model is not the only framework for describing unicellular interactions. Protistan populations, like prokaryotes, have also been studied in flow-through systems using input–output dynamics, including two-stage chemostat cascades for bacterivorous ciliates (Curds and Cockburn, 1971; Legner, 1973; Sambanis and Fredricson, 1987). The LV model is best suited to closed systems, where nutrient depletion and metabolite accumulation constrain growth, often requiring logistic fits that emulate metazoan population dynamics—an adjustment unnecessary in chemostat models.

Beatty et al. demonstrated the advantage of using large volumes of ocean water for the comparison of techniques estimating protistan phagotrophy. This way, they utilized the oligotrophic nature of ocean water masses allotting abundant volume of water to each protistan cell. Their approach reduced accumulating metabolites, which might otherwise impede population response. They compared results obtained using two techniques, the dilution method and fluorescently labeled bacteria (FLB) disappearance. In parallel field studies both methods substantially underestimated prey mortality. In laboratory experiments and field studies in the North Pacific Subtropical Gyre, grazing was found to dominate prey mortality, while viral lysis had only a minor additive effect. In contrast to laboratory results, direct comparison in field experiments performed in the North Pacific Subtropical Gyre off Hawaii resulted in an order of magnitude difference between grazer-mediated mortality rates using the two methods. These findings have important implications for how marine primary production is quantified and integrated into models of the higher levels of oceanic food webs.

Two more contributions address the central role of protists in the global carbon cycle. Much anthropogenic CO_2_ fixed in the deep oceans is captured by photosynthetic microbial eukaryotes, whose productivity is often limited by nitrogen and phosphorus availability. Coastal waters are often replete with N and P from human waste, but in the deep oceans where most CO_2_ is fixed, many of these photosynthesizing cells termed mixotrophs, supplement their diets with N and P from bacterivory to significantly increase their photosynthetic ability.

Many ocean protists cannot be grown in laboratory culture, so one of the few ways to know they exist is to take samples of transcriptomes (genomes assembled from mRNA transcripts) (Keeling et al., 2014), and ribosomal RNA genes from the oceans during transects by laboratory ships. Mining large-scale transcriptomic datasets, Romero et al. (2025) demonstrated that nearly 70% of oceanic protist diversity remains functionally uncharacterized, a major source of uncertainty in carbon-cycle models.

Using the MarPRISM machine-learning framework Thomas et al., studied transcriptomes of specialist photosynthesizers (autotrophs), specialist heterotrophs and mixotrophs sampled at varying depths and latitudes during four different oceanographic research cruises in the North Pacific during spring and summer seasons of 2015, 2017, and 2019. Samples were split into controls and nutrient experiments using N, P, and iron amendments. Complex patterns of relationships between trophic preference, light levels, depth, latitude and nutrient availability were reported.

Lin et al., studying coastal waters off northeastern Taiwan, identified several nonmotile chlorophytes as potential bacterivores, but also uncovered previously overlooked mixotrophic diversity by combining Lyso Tracker staining, flow cytometry, and sequencing approaches, further refining our understanding of microbial carbon flow under changing environmental conditions.

At the cellular level, predator–prey interactions can also drive evolutionary innovation. Moon et al. applied single-cell transcriptomics to examine interactions between the ciliate *Tetrahymena pyriformis* and *Vibrio cholerae*, identifying heterogeneous bacterial responses that may underlie both bacterial survival and predator mortality. Although environmental extrapolation is limited, the study introduces the MicroSplit approach as a powerful tool for dissecting microbial interactions within food webs.

Finally, Shen et al. used iRNA interference to study the genetic control of cilia in *Euplotes amieti* highlighting how single-celled eukaryotes, shaped by over a billion years of evolution, provide cost-effective and mechanistically informative models for fundamental cellular processes relevant to medicine. Cilia—once thought to serve only locomotory roles—are now recognized as sophisticated sensory and regulatory organelles (Valentine and Van Hout, 2021) that control phagotrophy. In addition to the uptake of external nutrients, phagotrophy is recognized to include autotrophy for the recycling of cellular infrastructure (Morelo et al., 2022; Germic et al., 2019), mitotrophy for the control of damaged or rogue mitochondria (Jeedigunta et al., 2021), and the re-purposing of microtubules to form the meiotic spindle when starvation is imminent in single celled organisms and sexual reproduction needs to be triggered to increase the genetic diversity of future generations. Given that ciliopathies underlie a growing number of human diseases as well as being central to such varied processes as brain development and function (Guo et al., 2015; Wang et al., 2024) and anterior/posterior embryonic development (Cole and Gaertig, 2022), insights from ciliated protists become increasingly valuable.

Together, the contributions in this Topic underscore phagotrophy as a unifying principle linking microbial ecology, global biogeochemical cycles, evolutionary biology, and human health.
